# Emotional Response to Humour Perception and Gelotophobia Among Healthy Individuals and Patients with Schizophrenia and Depression, with Signs of a High Clinical Risk of Psychosis

**DOI:** 10.17816/CP65

**Published:** 2021-03-20

**Authors:** Daria D. Volovik, Maria A. Omelchenko, Alyona M. Ivanova

**Affiliations:** Federal Centre of Brain and Neurotechnologies; Mental Health Research Center; Pirogov Russian National Research Medical University

**Keywords:** gelotophobia, the fear of being laughed at, emotion, facial expression, humour, risk of psychosis, attenuated positive symptoms, гелотофобия, страх насмешки, эмоции, выражение лица, юмор, риск психоза, аттенуированные позитивные симптомы

## Abstract

**Introduction.:**

Investigating early changes in the emotional sphere within the schizophrenia course is a perspective direction in clinical psychology and psychiatry. Intactness of positive emotions, in particular, humour perception, may be a very important resource for patients. At the same time, humour perception is very sensitive to pathological conditions, such as the fear of being laughed at, known as gelotophobia. Those with gelotophobia perceive laughter as dangerous, rather than pleasant, and they can hardly distinguish between teasing and ridicule. Gelotophobia was confirmed to be expressed among people with mental disorders. Nonetheless, knowledge relating to the fear of being laughed at, was mostly generated among the non-clinical samples.

**Objectives.:**

Thus, the aim of the study was to provide more clinical data on gelotophobia manifestations associated with schizophrenia spectrum disorders; the emotional response and facial expression of patients with gelotophobia were studied, in particular, regarding their perception of humour, including during the early stages of disorders, by comparison with healthy individuals.

**Methods.:**

n=30 controls and n=32 patients with schizophrenia and with depression with signs of a high clinical risk of psychosis took part. Two short videos, comic and neutral, were shown to the participants, while videotaping their facial expression, followed each by a self-reported measure of emotional responses. Participants also completed the State-Trait Anxiety Inventory, the PhoPhiKat30 and the Toronto Alexithymia Scale.

**Results.:**

Gelotophobia was significantly higher within the clinical group. It correlated with a lower frequency of grins among the patients during the comic video, while this was not the case in the control group. Gelotophobia was related to state and trait anxiety in both groups, but only in the clinical group did state anxiety increase after watching the comic video. Gelotophobia correlated with alexithymia and was twice higher among the patients compared to the controls.

**Conclusion.:**

Thus, gelotophobia has not only quantitative, but also qualitative specifics in patients with schizophrenia, and those with depression with signs of a clinically high risk of psychosis, compared to healthy controls.

## INTRODUCTION

According to many scholars, the recognition and expression of emotions that are the basis of nonverbal communication, reflect a decrease in the ability to process and apply social information, which leads to social incompetence [1]. These disorders are more common for schizophrenia spectrum disorders than affective disorders [2]. The socio-emotional deficit is also related to a poor functional outcome for patients, with a high clinical risk of psychosis [3]. Humour perception may be regarded as a strong marker of emotional expression disorder or intactness. Patients with schizophrenia exhibited significant and substantial deficits in humour recognition, compared to the patients with depression and anxiety [4,5], while patients with affective disorders demonstrated a greater decrease in laughter expression, compared to those with schizophrenia spectrum disorders and the healthy controls [5]. All the aforementioned groups of patients have difficulties in relation to humour comprehension [6]. The inability to orient in social interactions involving humour and laughter, may lead to negative emotional reactions to humour, including an increased fear of being laughed at – gelotophobia [7].

Gelotophobia is defined as the pathological fear of becoming an object of ridicule, initially regarded as a form of social phobia [7]. Firstly, descriptions of gelotophobia were presented by a psychotherapist, M. Titze, based on his single-case observations in clinical practice [7,8]. Later, the concept was developed within a psychometrical approach. W. Ruch and R.T. Proyer used prototypical statements of individuals with gelotophobia, collected from clinical practice, to elaborate on the first self-reported gelotophobia scale - the Geloph [9]. Using this first version of the questionnaire they empirically separated a group of clinically-diagnosed gelotophobic patients (provided by M. Titze) from the groups of shame-based and non-shame-based “depressed neurotics”, as defined by Nathanson [10], and normal controls [11]. Subsequently, the gelotophobia scale has been revised several times [12], and the modern instrument, the PhoPhiKat<30> includes two additional gelotophobia subscales, such as gelotophilia (the joy of being an object of laughter) and katagelasticism (the joy of laughing at others) [13]. From this point, the concept of the fear of being laughed at, became an area of interest, and has been studied in many countries and in many languages. In a multi-national study by R. Proyer et al. the data from 73 countries and 42 languages were analysed altogether [14].

Gelotophobia has maladaptive characteristics: conviction in one’s own ridiculousness, perception of laughter as a threat, increased anxiety and shame, stiffness and timidity, sensitivity and social isolation in extreme cases [15-17]. Gelotophobic people are very observant in social situations and become easily suspicious of the laughter of others. They can hardly distinguish between happy, joyful and derisive kinds of laughter, and cannot experience laughter as relaxing or positive, only as a means of aggression. They tend to interpret even benevolent or neutral kinds of humour-related situations as threatening [15]. Among the general population, the frequency of gelotophobia ranges from 5% to 12% in different countries, and from 7% to 15% in Russia [16-18].

The first data regarding the emotional response and expression of people with gelotophobia were provided by W. Ruch et al. [15]. They discovered that people with gelotophobia automatically respond to smiling and laughing faces with a facial expression of contempt, rather than the more natural and normative reaction of smiling back. Gelotophobic people also tend to perceive others’ smiles as less joyful and more contemptuous; they do not experience positive emotions watching smiling faces, in the same way as other people [19]. Thus, gelotophobia may not only distort the perception of the target of laughter and the motives of laughter, but also constitutes an emotional response to humour in general, in a wide range of humour-related situations.

M. Titze discussed gelotophobia in relation to sociophobia and shame-bound anxiety, although regarded it as a relatively independent phenomenon [8]. Recent empirical studies have confirmed the high correlation between the fear of being laughed at, and social anxiety [20-22]. Gelotophobia also occurs more often in patients with avoidant personality disorder, moreover, all patients with both social anxiety and avoidant personality disorder were also defined as gelotophobic [21]. Based on this, the fear of being laughed at was regarded as a possible additional diagnostic criterion for these disorders.

A number of clinical studies confirmed a higher expression of gelotophobia amongst those with various mental disorders [21-24], including schizophrenia spectrum disorders [23,25,26]. Nonetheless, knowledge relating to the fear of being laughed at was mostly generated in relation to the samples of individuals without clinical diagnoses, within the frame of individual differences [15,19,27], and there is still a lack of clinical data.

Despite the continuous discussion relating to the distinction between the fear of being laughed at as a trait and as a pathological condition, the qualitative specifics of gelotophobia among those with severe mental disorders have not been sufficiently studied.

The aim of this study was to provide more clinical data on gelotophobia manifestations in schizophrenia spectrum disorders. We studied the emotional response and facial expressions regarding the perception of humour among inpatients with schizophrenia spectrum disorders, depressed patients with a high risk of psychosis and healthy controls.

The hypotheses of the study were the following: 1) the emotional response to humour differs in patients with schizophrenia spectrum disorders compared to the controls; 2) these peculiarities differ depending on the level of psychopathology (schizophrenia versus depression with a high clinical risk of psychosis); 3) the peculiarities of the emotional response to humour in patients with schizophrenia spectrum disorders, can be attributed to gelotophobia.

## MATERIAL AND METHODS

1) The stimulus video material consisted of two clips – the comic and the neutral clips. The videos were compiled from short clips taken from the YouTube platform. Fragments of each video were selected in such a way, so as to be very similar in terms of duration, brightness, quality, as well as format (only horizontal orientation). Each of the two videos lasted around three minutes.

- The comic video did not have a storyline. It consisted of amusing clips about dogs (for example, a dog dancing to music; a dog walking in boots, etc.). It was humorous in terms of content, aimed at forming positive emotions.
- The neutral video consisted of short clips about dogs’ lives (for example, a dog being walked; a dog in the process of being trained, etc.); it did not have a storyline and was supposed to be emotionally neutral.

Participants watched the neutral video first and then the comic video, while their facial expression was videotaped and later analysed. Emotional (laughter) expressions were categorized as none, smile (with no vocalization), grin (a smile with a short-term and slight vocalization, the mouth is mostly closed), laughter (open mouth, obvious vocalization), burst of laughter (loud vocalization, body movements).

After each video, the participants evaluated their subjective emotional response. Both before and after watching the comic video, they also completed the State Anxiety Inventory [28,29] in order to measure the potential anxiety evoked by the humorous stimuli. After the whole experiment, participants were assessed in relation to the Trait Anxiety Inventory [28,29] and the Toronto Alexithymia Scale [30,31] in order to control possible alternative or additional factors of emotional distortions.

As one can see, the chosen humorous stimuli were simple and benevolent, or at least neutral, and could hardly evoke an idea of negative intent or emotions – at least in healthy participants. Therefore, as a result of such a method we suggested accessing an emotional response to humour.

2) An emotion evaluation scale, developed by the authors, with a written list of 10 emotions (joy, delight, grief, anxiety, sadness, fear, anger, indifference, shame, disgust) was presented to the participants after each video. The list was created on the basis of P. Ekman’s classification of basic emotions [32].

Indifference, delight, shame and anxiety were added to the list because of their association with the variables that were focused on, namely, gelotophobia, alexithymia, personal and situational anxiety. Participants were to choose the emotions they experienced while watching the stimulus video, and to evaluate their intensity from 1 to 5.

3) The PhoPhiKat <30> was developed by W. Ruch and R. Proyer [13]. The Russian adaptation was proposed by E.M. Ivanova et al. [18].

PhoPhiKat <30> consists of 30 items. The questionnaire assesses gelotophobia (the fear of being laughed at), gelotophilia (the joy of being an object of laughter) and katagelasticism (the joy of laughing at others); the last two subscales were not used in the present study.

The participants were to rate each of the statements on a four-point Likert scale (from “completely disagree” to “fully agree”).

4) We also used the Scale of Prodromal Symptoms (SOPS) [34] to assess attenuated prodromal symptoms, the Positive and Negative Syndrome Scale (PANSS) [33] to assess psychotic symptoms and the Hamilton Depression Rating Scale (HDRS) [35] to assess depressive symptoms.

The SOPS forms part of the Structured Interview for Prodromal Syndromes (SIPS). It may be conceptualized as analogous to the PANSS for patients who are not fully psychotic (at a high clinical risk of psychosis). The SOPS contains four subscales for positive, negative, disorganized and general symptom constructs. Attenuated positive symptoms were assessed on the positive subscale of the SOPS.

The PANSS is one of the best-validated instruments for measuring the symptom severity of patients with schizophrenia, that we used to assess patients with first psychosis in this study.

The HDRS is a 21-item depression rating scale for determining a level of depression in patients with first psychosis and with signs of a high clinical risk of psychosis.

All patients were examined according to these scales twice: firstly, at the point of admission and secondly, after completion of the main course of therapy, before being discharged from the hospital.

A psychological study was carried out at the second stage to identify any emotional disturbance among patients with psychosis and at a high clinical risk who were close to remission.

All subjects gave their informed consent for inclusion before they participated in the study. The study was conducted in accordance with the Declaration of Helsinki, and the protocol was approved by the Ethics Committee of the Mental Health Research Center on 05.05.2016 (project identification code 281).

The following statistical methods were used in the quantitative analysis of the data: the Mann-Whitney criterion, the Wilcoxon signed-rank test and the Spearman's rank correlation coefficient.

### Participants

In total, 62 participants took part in the study. The control group consisted of 30 conditionally healthy individuals (19 women, 11 men) at the age of 22.9±5.7.

The clinical group consisted of 32 patients (all men) between the ages of 18 and 24 (M = 19.6 years, SD = 2.04) hospitalized at the Mental Health Research Center (MHRC) and divided into two subgroups:

#### Subgroup 1

(n = 16, at an age of 20.8±2.3) consisting of primary inpatients, hospitalized with the first depressive episode (F32) with signs of a high clinical risk of psychosis [36], which have been identified according to the SIPS [37] as Brief Limited Intermittent Psychotic Symptoms (BLIPS) and Attenuated Positive Symptoms (APS) [38]. The mean score recorded by the SOPS was 45.1±10.6 and the mean score recorded by the HDRS was 26.5±6.2, being 24.2±10.9 and 6.8±1.1 at the time of admission and at the second stage before discharge, respectively.

#### Subgroup 2

(n = 16, at an age of 21.6±1.6) with the first episode of psychosis, with diagnoses of F20 (three patients) and of F25 (13 patients). The mean score recorded by the PANSS at the first stage was 86.3±12.8, the mean score recorded by the HDRS was 20.2±8.6, and at the second stage, the scores were 53.6±13.5 and 6.1±1.8, respectively.

All patients showed significant clinical improvement, assessed by the scales SOPS, PANSS and HDRS after the reduction of the leading syndrome, before being discharging from the hospital. Thus, in patients with signs of a high clinical risk for psychosis, depressive symptoms were reduced (HDRS<8), which could otherwise influence the results of the study. All diagnoses, as well as assignment to the clinical subgroup, were verified by the psychiatrists. The patients took medication, which included atypical antipsychotics (risperidone, quetiapine, olanzapine) of an average dosage, converted to chlorpromazine equivalents [39] namely 292.5±206.1 mg per day in group 1 and 611.7±209.2 mg per day in group 2, as well as selective serotonin reuptake inhibitors (SSRIs), (fluvoxamine, sertraline, paroxetine). In order to exclude the side effects of medical treatment, that could influence the data, all patients were examined with the UKU (The UKU Side Effects Rating Scale for the Registration of Unwanted Effects of Psychotropics) [40], and none of them revealed any significant unwanted effects (points per item were 0 – no side effects or 1 – mild side effects that do not interfere with the patient's performance).

### Procedure

The procedure included several stages. At the first stage, stimulus videos (first the neutral, then the comic video) had been shown to the control group of healthy individuals. The participants’ emotional expression while watching the video was recorded with a Logitech C910 camera for further data processing. All the participants had been informed of being recorded and signed an informed consent, agreeing to their participation in the study.

After watching each video, the participants evaluated their emotional state according to the list of 10 emotions. Participants had to choose the emotions they experienced while watching each video, and evaluate their intensity from 1 to 5 (1 - low; 2 - moderate; 3 - above average; 4 – fairly high; 5 - high).

The Trait Anxiety Inventory was administered to the participants immediately before and immediately after watching the comic video, in order to assess an increase in anxiety in relation to humour perception.

At the second stage, participants were examined by the STAI (trait anxiety), the PhoPhiKat and the TAS scales. The cut-off point of 2.5,12 was applied, in order to distinguish participants with gelotophobia from those with no fear of laughter.

## RESULTS

### Expressive reactions

Mean rank comparisons, using the Mann-Whitney criterion showed, that in the control group, the subjects significantly more often smiled (U = 196.500; Z = -3.784; p = 0.0001), grinned (U = 265.500; Z = -3.433; p = 0.001) and laughed (U = 330.000; Z = -3.004; p = 0.003) while watching the comic video, compared to the neutral video; no one laughed while watching the neutral video, which made it possible to assume the validity of the stimulus material (see Table 1). The expressive reactions of all the patients were, in general, significantly poorer compared to the control group, according to the results of the Mann-Whitney criterion. The differences between the clinical and the control group were statistically significant: the patients smiled, grinned and laughed less (p < 0.05) in relation to the comic video, and smiled even less when watching the neutral video (p = 0.001). No differences were found between the clinical subgroups. 

**Table 1:** Table 1. Frequencies of smiles, grins and laughter in the groups

	Control group (n=30)		Clinical group (n=32)	
	neutral video	comic video	neutral video	comic video
n, smiles	70	201	28	99
n, grins	3	57	0	10
n, laughter	0	22	0	7

Moreover, unlike the control group, the comparison between the two videos only showed significant differences with regard to smiles (U = 64.500; Z = -2.651; p = 0.015) for the group suffering from depression with signs of a high clinical risk and no significant differences for the psychotic group. In the latter group, there was no laughter at all and only one person grinned twice during the comic video.

### Self-reported emotional reactions

An analysis of self-reported emotional reactions after each video, using the Mann-Whitney criterion demonstrated increased joy (U = 232.500; Z = -3.303; p = 0.001), delight (U = 240.000; Z = -3.706; p = 0.0001) and surprisingly, sadness (U = 375.000; Z = -2.313; p = 0.021) after watching the comic video, compared with the neutral video in the control group. In contrast, no significant differences between emotional reactions were found in each of the clinical groups. Patients in the psychotic group tended to report higher levels of delight after watching the comic video, rather than the neutral video, but this result is not particularly significant (U = 82.000; Z = -1.955; p = 0.086).

### Gelotophobia

Mean rank comparison by the Mann-Whitney criterion revealed increased gelotophobia, measured by the PhoPhiKat, among the patients than the control group (25.78 and 36.86 relatively, U = 308.500; Z = -2.420; p = 0.016), which confirmed our hypothesis. The pattern was the same for each of the clinical subgroups: the level of gelotophobia was greater in the group with depression with signs of a high clinical risk (p = 0.032) and in the psychotic group (p = 0.015), than in the control group. At the same time, no differences were found between the subgroups of patients.

Next, we examined the correlations between the level of gelotophobia, expressive reactions (frequencies of smiles, grins and laughter) and emotions, reported by the participants after each video, using Spearman’s criterion. In the control group, gelotophobia was not related to the frequency of smiles and laughter in relation to the videos, neither was it associated with any of the emotions. On the contrary, in the clinical group, a higher level of gelotophobia was associated with a lower frequency of grins while watching the comic video (r = -0.466; p = 0.007). More detailed analysis revealed that in the group with depression with a high clinical risk, gelotophobia correlated with the frequency of smiles while watching the neutral video (r = -0.592; p = 0.016) and grins while watching the comic video (r = -0.576; p = 0.020). At the same time, in the psychotic group there were no such correlations (r = 0.024; p = 0.929 and r = -0.281; p = 0.292, relatively). No correlations were found in any of the groups between gelotophobia and reported emotions after watching the videos.

### Gelotophobia and anxiety

Gelotophobia correlated positively to trait anxiety, measured by the STAI in both groups by the Spearman criterion. Higher gelotophobia was related to a higher level of trait anxiety in the control group (r = 0.677; p = 0.0001) and in the clinical group (r = 0.580; p = 0.001), with the same pattern for each of the subgroups.

Differences in state anxiety before and after watching the comic video were analysed for each of the groups, using the Wilcoxon criterion. In the control group, state anxiety between the two stages did not differ (mean ranks 12.11 and 16.04; Z = -0.471; p = 0.638), while in both clinical subgroups, the level of anxiety increased after watching the comic video (mean ranks 7.25 and 8.12; Z = -2.586; p = 0.01 for the group with depression with a high clinical risk, and mean ranks 1.75 and 8.96; Z = -3.210; p = 0.001 for the psychotic group).

Then we calculated the numerical difference between the score before and after watching the comic video. The increase of this parameter reflected increase of state anxiety and it appeared to be associated with gelotophobia in the control group (Spearman criterion, r = 0.471; p = 0.009) as well as in the clinical group (r = 0.422; p = 0.016). The pattern in the subgroups was the same, although in the group with depression with a high clinical risk, the correlation did not reach the level of significance (r = 0.461; p = 0.072), while in the group of psychotic patients the level of significance was reached (r = 0.520; p = 0.039).

### Gelotophobia and alexithymia

**Figure 1:**
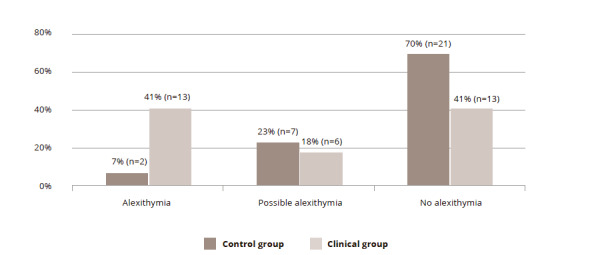
Figure 1. The level of alexithymia in the groups

Not surprisingly, the Mann-Whitney test revealed that higher levels of alexithymia, measured by the Toronto Alexithymia Scale, were more common among the patients than the healthy participants (mean ranks 1240 and 713, respectively, W = 248.000; Z = -3.270; p = 0.001). Figure 1 demonstrates the distribution of the alexithymia levels in the two groups.

Gelotophobia correlated with alexithymia in both groups, but in the clinical group, the Spearman coefficient was almost twice higher (r = 0.746; p = 0.0001) than in the control group (r = 0.490; p = 0.006).

## DISCUSSION

In the group of healthy participants, the comic video produced more emotional expressions (smiles, grins and laughter), and higher levels of joy and delight than the neutral video, which is in line with our expectations and confirmed the validity of the stimulus material. Unexpectedly, healthy people also reported higher levels of sadness in relation to the comic video, which is hard to interpret. Perhaps this was related to the subjects’ assessments of the humour quality, which always seem to be relatively low in experimental, laboratory conditions.

The expressive responses of all the patients were significantly poorer compared to the controls, with no differences revealed between the clinical subgroups, which is consistent with a number of studies regarding less emotional expressivity among those with schizophrenia spectrum disorders [41,42]. Probably because of this, the expressive reactions of the patients differed between the neutral and comic video only in terms of the frequency of smiles in the group with depression with a high clinical risk, but not with regard to grins and laughter. The patients with psychotic disorders did not exhibit any differences at all.

An analysis of the self-reported emotional reactions in both groups of patients revealed no differences after watching the comic or the neutral video, by contrast with the control group, which reflected a deficit not only in relation to expressivity, but also in the subjective, emotional experience.

Gelotophobia was significantly higher among the group of inpatients than the control group, which is consistent with the data of previous studies [23,26]. At the same time, no differences were found between the clinical subgroups.

Neither the expressive laughter reactions, nor the emotions experienced with regard to the comic video were associated with gelotophobia in the control group, which seemed to contradict the data of Ruch et al. [27]. However, it is worthy of note, that the humour chosen for the present study was far removed from social interaction and thus, the danger of being laughed at did not prevent the participants from being amused by the comical situations, even in the case of healthy participants with a greater fear of being laughed at as a trait. On the contrary, in the group with depression with a high clinical risk, gelotophobia correlated negatively with the frequency of grins while watching the comic video, and surprisingly, with the frequency of smiles while watching the neutral video. This could reflect the tendency of this group to control their reactions in situations related to the context of humour and laughter, even innocent situations, as was the case in the present study, which is relatively consistent with the concept of gelotophobia. This control could also be expanded to include more neutral, social situations. Unexpectedly, in the psychotic group, gelotophobia did not correlate with expressive reactions to the comic video. Possibly, this was due to deeper disturbances in emotional expressivity among these patients, unrelated to gelotophobia. Nevertheless, this result needs to be addressed in future studies.

No correlations in any of the groups were found between gelotophobia and reported emotions after watching the videos. Thus, the comic video did not result in more fear, shame, anxiety or anger, as one might hypothesize. It is worthy of note, however, that the scale of emotions was a self-reported measure, therefore, could be more influenced by the tendency to control oneself and to reveal more socially desirable results.

Gelotophobia was associated with trait anxiety in all the groups with a particularly significant connection in the control group. At the same time, watching the comic video increased state anxiety among the patients only, while this did not differ among the control group. Gelotophobia correlated with an increase in state anxiety relating to the comic video in all the groups: the higher the gelotophobia, the higher the increase in anxiety. However, in the group with depression with a high clinical risk, the connection did not reach a level of significance. Thus, the perception of humour and laughter, even regarding such innocent and safe topics as pets’ humour, evoked an increase in anxiety among those with gelotophobia.

As expected, gelotophobia was related to the level of alexithymia in both groups, but in the clinical group, it was almost twice higher than in the control group. Thus, the difficulty of understanding and expressing one’s own emotions, as well as understanding the feelings of others, could be one of the psychological mechanisms underlying gelotophobia among these patients.

Overall, the results led to the conclusion that gelotophobia in mentally ill people, in particular, those suffering from schizophrenia spectrum disorders, has specific differences, compared to the fear of being laughed at among healthy individuals. The differences are not just quantitative, but also qualitative, and they may crucially distort humour and laughter perception, along with the behavioural reaction to humour in these patients.

## CONCLUSIONS

As expected, both patients with schizophrenia and depression with signs of a high clinical risk of psychosis, had a lower emotional expression to humour perception compared to the controls. Similarly, the patients showed no emotional reaction to the comic content, compared to the neutral content.

Consistent with earlier data, gelotophobia was significantly higher among patients with schizophrenia spectrum disorders, compared to the healthy controls. The fear of being laughed at, correlated with a lower frequency of grins among the patients in relation to the comic video, while among the controls this reaction was not in evidence.

Gelotophobia was related to trait anxiety in both groups, but only in the clinical group was it associated with increased state anxiety, measured both before and after watching the comic video. Thus, the study provides evidence that humour perception, even of an innocent nature, may evoke anxiety among patients with schizophrenia spectrum disorders, which is related to gelotophobia.

Unsurprisingly, alexithymia was higher among the patients, and gelotophobia was associated with it. Nonetheless, it is interesting that this association was twice higher among patients compared to the controls.

Thereby, gelotophobia has not only quantitative, but also qualitative specifics in patients with schizophrenia spectrum disorders, compared to healthy controls, and it is related to an emotional response to humour perception.

### Limitations

The present study has several limitations. Firstly, due to organizational issues, the clinical group consisted only of male participants. Further research with female patients is needed to clarify possible gender differences. Secondly, the study lacked technical equipment, for example, with the help of specialized computer programs it could be possible to register the facial expression of the participants more accurately. Thirdly, all the patients were assessed after antipsychotic treatment and, despite the low intensity of the side effects, the higher dosage in patients with first psychosis could also influence the difference between groups.

## Authors contribution

Daria D. Volovik: literature review, conceptualization, data collection, data analysis, writing original draft of the paper; Alyona M. Ivanova: literature review, conceptualization, methodology, data analysis, writing – review and editing, supervision, project administration; Maria A. Omelchenko: literature review, conceptualization, writing – review and editing, project administration.

## Conflict of interests

The authors claim no conflict of interests.

## Funding

This research received no external funding
